# A Randomized, Double-Blind, Placebo-Controlled Trial to Determine the Effectiveness of a Polyphenolic Extract (*Hibiscus sabdariffa* and *Lippia citriodora*) for Reducing Blood Pressure in Prehypertensive and Type 1 Hypertensive Subjects

**DOI:** 10.3390/molecules26061783

**Published:** 2021-03-22

**Authors:** Javier Marhuenda, Silvia Pérez-Piñero, Raúl Arcusa, Desirée Victoria-Montesinos, Fernando Cánovas, Maravillas Sánchez-Macarro, Ana María García-Muñoz, María Querol-Calderón, Francisco Javier López-Román

**Affiliations:** 1 Faculty of Health Sciences, San Antonio Catholic University of Murcia (UCAM), 30107 Murcia, Spain; sperez2@ucam.edu (S.P.-P.); rarcusa@ucam.edu (R.A.); dvictoria@ucam.edu (D.V.-M.); fcanovas@ucam.edu (F.C.); msanchez4@ucam.edu (M.S.-M.); amgarcia13@ucam.edu (A.M.G.-M.); mquerol@ucam.edu (M.Q.-C.); jlroman@ucam.edu (F.J.L.-R.); 2 Primary Care Research Group, Biomedical Research Institute of Murcia (IMIB-Arrixaca), 30120 Murcia, Spain

**Keywords:** *Hibiscus sabdariffa*, *Lippia citriodora*, polyphenol, blood pressure, hypertension

## Abstract

Hypertension is an important factor of cardiovascular diseases and contributes to their negative consequences including mortality. The World Health Organization estimated that 54% of strokes and 47% of cases of ischemic heart illness are related to high blood pressure. Recently, *Hibiscus sabdariffa* (HS) and *Lippia citriodora* (LC) have attracted scientific interest, and they are recognized for their high content of polyphenols as these may prevent several disease factors, such as hypertension. The aim of the present study is to determine if supplementation with an HS-LC blend (MetabolAid^®^) may be effective for the treatment of type 1 hypertensive sedentary populations. A total of 80 type 1 hypertensive subjects of both sexes were included in the study and were treated with placebo or the HS-LC extract, and both groups were treated over 84 days. The blood pressure (diastolic, systolic, and pulse pressure) was measured throughout the day, for each of the days of the study duration and determined using Ambulatory Blood Pressure Monitoring (ABPM). Physical activity was determined throughout the study to ensure similar conditions related to exercise. The results showed the capacity for reducing the blood pressure parameters in the case of the HS-LC extract. The daily consumption of the HS-LC extract but not the placebo over 84 days was able to reduce the daytime parameters related to blood pressure. The most remarkable results were observed in the measurements performed during the daytime, especially in the systolic blood pressure showing statistically significant variation.

## 1. Introduction

Hypertension (HT) has become a major cause of mortality worldwide. An estimated 691 million people worldwide suffer from high blood pressure [1]. Of the 15 million deaths caused by circulatory diseases, 7.2 million are due to coronary heart disease and 4.6 million to brain vascular disease [2]. The prevalence of HT was 31.1% in adults worldwide in 2010 [3]. In the United States, an estimated 50 million patients have HT [4], and some 60,000 deaths annually are caused directly by HT [5].

The World Health Organization estimated that 54% of strokes and 47% of cases of ischemic heart illness are connected with high blood pressure. This is due to the main risk factors for cardiovascular morbidity and mortality as well as HT [6]. The decrease of strokes in recent years is a consequence of the reduction in blood pressure [7]. However, while the connection between high blood pressure and cardiovascular morbidity and mortality is very clear [5], blood pressure is frequently inadequately controlled. Either the blood pressure is not measured, the physician fails to react in the face of elevated blood pressure values [8], treatment is not provided optimally, or the patient fails to take the necessary medication regularly [9].

HT is a multifactorial disease that is strongly associated with lifestyle and especially with the consumption of alcohol, foods high in salt and fat, a sedentary lifestyle, and obesity among others [5]. Tobacco, despite not constituting a risk factor for hypertension, can acutely increase the heart rate and blood pressure, aggravating cardiovascular problems in hypertensive patients. HT can be prevented by modifying these risk factors from an early age, and it is necessary to take actions that reduce exposure to these factors, thus, reducing the incidence of cardiovascular diseases, including HT [5].

Polyphenols are natural extracts that have been extensively studied over recent years due to their antioxidant, antihypertensive, and anti-inflammatory capacities, in addition to their possible role in the prevention and management of several diseases, such as cardiovascular diseases, HT, diabetes, cancer, and neurodegenerative diseases [10,11,12]. Recently, *Hibiscus sabdariffa* (HS) and *Lippia citriodora* (LC) have attracted scientific interest for their potential use in the treatment of metabolic syndrome [13]. In particular, HS and LC exert a potent antihypertensive capacity [14,15].

Previous studies using a product called MetabolAid^®^, comprised of HS and LC extracts highly purified in anthocyanins and verbascoside polyphenols, respectively, previously reported a significant hypotensive effect, albeit using single blood pressure measurements in placebo-controlled clinical trials. Overweight and obesity are associated with higher blood pressure figures. Several studies, including the Framinghan study, have shown the high prevalence of hypertension in the obese population. Similarly, research demonstrated that weight loss with a hypocaloric diet in obese patients was associated with reductions in blood pressure levels and reductions in the pharmacological therapy necessary to keep it under control [16,17].

The basis of the present work is the use of MetabolAid^®^ as part of antihypertensive treatment, due to the limited number of clinical studies linking LC and HS polyphenols with hypertension. Both plants that contain the ingredient are recognized for their high content of polyphenols, powerful antioxidant molecules that may be useful in many diseases such as HT, oxidative stress, dyslipidemia, lipid mobilization, and endothelial stiffness [10]. The aim of the present study is to determine if supplementation with HS-LC extract is useful for the treatment of pre-hypertension and type 1 hypertensive, non-medicated populations.

## 2. Results

The characteristics of the volunteers of the study are displayed in Table 1. To verify that the randomization was correct, and to be able to control the evolution of the volunteers in a suitable approach, the blood pressure was initially determined using a sphygmomanometer (Table 1). As can be seen, the systolic blood pressure (SBP) showed similar values between groups, leading to 133.64 ± 12.42 mmHg in the control group and 135.43 ± 1.44 mmHg showed by the experimental group (*p* = 0.516). Likewise, the diastolic blood pressure (DBP) also showed similar values when comparing the control and experimental groups, leading to 86.13 ± 7.6 mmHg and 90.27 ± 8.05 mmHg, respectively.

### 2.1. Blood Pressure Measured with Ambulatory Blood Pressure Monitoring

The determination of the blood pressure with ambulatory blood pressure monitoring (ABPM) allowed for multiple interpretations of the results, leading to reliable parameters that reflect the cardiovascular capacity.

#### General SBP, DBP, and Mean Blood Pressure (MBP)

**Figure 1:**
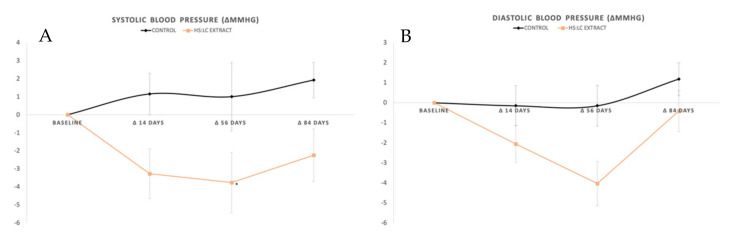
(**A**) Systolic blood pressure and (**B**) diastolic blood pressure determined using ambulatory blood pressure monitoring (ABPM). Represents statistically significant intragroup differences. Values are expressed by the increase (in mmHg) between the baseline and each control day.

Figure 1 shows the evolution of both the DBP and SBP measured by ABPM. For the SBP, the intake of the HS-LC extract reduced the initial parameters (126.4 ± 1.8 mmHg), particularly at day 56 (−3.76 ± 10.13 mmHg; *p* < 0.05). This was not observed for the placebo, which showed similar values throughout the whole study (*p* > 0.05). The comparison between the placebo and HS-LC groups along the whole study led to significant differences in the SBP (*p* < 0.02). Therefore, the HS-LC extract appeared to improve the SBP to a greater extent compared with the placebo, which was observed from the first 56 days of treatment.

In contrast, the DBP remained stable throughout the whole study regardless of the HS-LC extract or placebo consumption (Figure 1). Therefore, neither the placebo nor HS-LC extract was able to change the DBP after 84 days of treatment. The variation of the mean blood pressure (MBP) along the study is shown in Figure 1. As can be seen, the values from both groups were similar at the baseline leading to homogeneity between groups. Due to the nature of MBP and considering the DBP values described, no statistically significant changes were observed during the study, despite the reduction in SBP values after the intake of the HS-LC extract (*p* = 0.19). 

### 2.2. Daily Evolution of Blood Pressure Measured with ABPM

The cardiovascular system is strongly influenced by circadian patterns. The incidence of cardiac events is clearly associated with daytime hours [18].

#### 2.2.1. Daytime SBP and DBP

**Figure 2:**
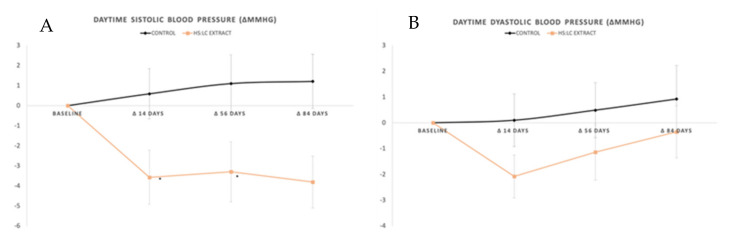
(**A**) Systolic blood pressure and (**B**) diastolic blood pressure determined using ambulatory blood pressure monitoring (ABPM). Represents statistically significant intragroup differences. Values are expressed by the increase (in mmHg) between the baseline and each control day.

The comparison between both the HS-LC and placebo groups revealed significant differences when the measurements of the daytime hours of the day were selected, as can be observed in Figure 2 if data are compared.

The HS-LC extract was able to reduce the daytime SBP but the daytime DBP remained at similar values as with the baseline (*p* < 0.29). Remarkably, the treatment with placebo did not lead to variations on the systolic or DBP (*p* > 0.05).

From a wide point of view, MBP determined for the daytime maintained similar values from baseline to day 84 in both the placebo and HS-LC groups (*p* > 0.05). The treatment with placebo was not able to reduce either the daytime systolic or DBP. However, the HS-LC extract appeared to be effective in reducing daytime SBP.

#### 2.2.2. Nocturnal SBP and DBP

**Figure 3:**
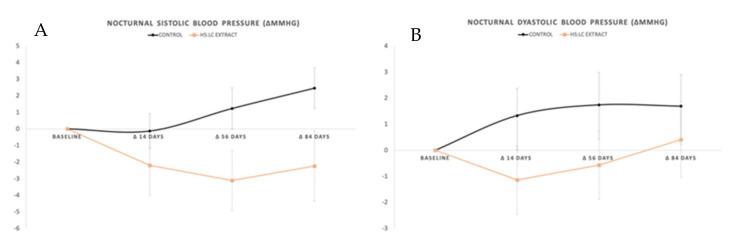
(**A**) Systolic blood pressure and (**B**) diastolic blood pressure determined by ABPM. Values are expressed by the increase (in mmHg) between the baseline and each control day.

The monitorization of nocturnal SBP throughout the study revealed an increase in the subjects treated with placebo (+2.46 ± 1.23 mmHg at day 84) and a decrease in those consuming the HS-LC extract (−2.24 ± 2.11 mmHg at day 84). However, the changes were not statistically significant in any case (*p* > 0.05). Similarly, placebo-controlled group showed a higher DBP (*p* > 0.05) at day 84 compared with at the baseline (+1.69 ± 1.21 mmHg). Therefore, no relevant changes were observed (Figure 3).

#### 2.2.3. Dipper

**Figure 4:**
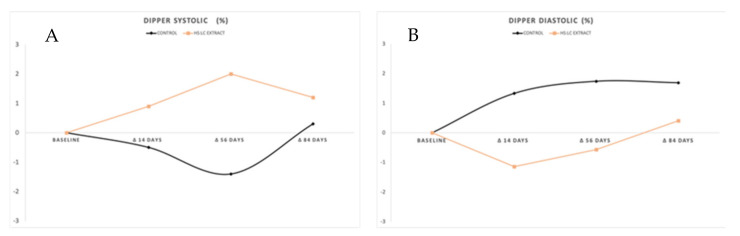
(**A**) Systolic blood pressure and (**B**) diastolic blood pressure determined by ABPM. Values are expressed by the increase (in mmHg) between the baseline and each control day.

Dipper values assessed using ambulatory blood pressure monitoring were not modified after product or placebo ingestion (Figure 4). Under normal conditions, a 10% decrease in the diurnal blood pressure compared to the nocturnal blood pressure is expected. After ingestion of the product, we did not observe that it induced a modification of this parameter.

### 2.3. Blood Parameters

As shown in Table 2, the blood parameters showed similar levels both at the baseline and at the end of the study (*p* > 0.05). There was a downward trend for the cholesterol and LDL values after the consumption of the product under study; however, the HS-LC extract did not improve (*p* > 0.05) the lipid profile of the subjects. The evolution of the lipid profile was similar (*p* > 0.05) between the subjects who consumed the placebo and those who consumed the HS-LC extract (Table 2). As observed for the lipid profile, the glycemic parameters showed similar values throughout all the study, regardless of the consumption of the placebo or the HS-LC extract (Table 2).

### 2.4. Physical Activity

Changes that may occur due to intrapersonal variation regarding physical activity can lead to errors in the interpretation of the results obtained [19]. The measurement of the METs by the accelerometer revealed that subjects from both the placebo and experimental groups maintained the same physical activity during the 84 days of the study. Regarding the placebo group, the values ranged (*p* = 0.418) from 1.7 ± 0.3 MET at the baseline to 1.8 ± 0.3 MET at the end of the study. The experimental group showed similar values (*p* = 0.842) both at the baseline (1.7 ± 0.4 MET) and at the end of the study (1.7 ± 0.3 MET).

Therefore, during the 84 days of treatment with the placebo or the HS-LC extract, the subjects maintained similar physical activity. This supports that the changes observed in subjects consuming the HS-LC extract were a consequence of the consumption and not a consequence of modified physical activity habits.

## 3. Discussion

The consumption of HS-LC extract significantly decreased the SBP compared to the control group, especially during the daily measurements. Habitually, the calyxes of HS have been used as a hypertension treatment [20]. In fact, animal and human studies have reported a reduction of SBP and DBP [20,21]. Physical exercise stimulates skeletal muscle adaptations as increased mitochondrial density, increased oxygen availability, increased blood flow, and distended arteries, which results in a lower resting heart rate and blood pressure and increased BMR. Studies hypothesized that bioactive compounds from the HS-LC extract could simulate the effects of physical exercise on the heart rate and blood pressure [17,20]. 

A study with 54 moderate hypertensive patients (similar to the present study) treated with sour HS tea revealed a noticeable reduction in blood pressure (systolic by 11.2% and diastolic by 10.7%). However, the length of the treatment (12 days) was not sufficient to draw firm conclusions [22]. A study from 2004 with 75 subjects reported that the treatment with an HS aqueous extract was sufficient to reduce the blood pressure by more than 10%. HS was reported to be safe, compared with 25 mg of captopril and 10 mg of lisinopril [23,24]. However, the comparison of the mentioned studies is difficult due to the poor standardization of the products used in previous clinical interventions.

Natural products, such as plant derivates are characterized by the presence of high content of polyphenols [25] with proven antihypertensive properties. However, the mechanism of action has not yet been accurately described [26,27]. The classic explanation of hypertensive properties of polyphenols was related to their antioxidant capacity, which reduces the formation of atheroma plaques. 

Scientific evidence has shown that certain plant-derived extracts, such as HS and LC, can modulate different metabolic pathways and activate the AMPK pathway favoring lipolysis and, therefore, fat loss [17], which could explain a decrease in blood pressure. HS and LC have shown therapeutic effects in oxidative stress treatments, lipid profile regulation, and the reduction of high blood pressure or atherosclerosis. That capacity may be attributed to their polyphenolic content and their capacity to inhibit low-density lipoprotein oxidation and decrease the atherosclerotic process [28].

The SBP was reduced, which may be explained by the vasodilator capacity of polyphenols [29]. However, the higher presence of a certain type of polyphenol and the differences in their chemical structure appeared to influence their bioavailability and bioactivity, which can, therefore, determine the final hypotensive effect. For example, the anthocyanin ponidine and certain flavonoids, such as cyanidin, catechin, and epicatechin, were able to generate apocynin-analogous compounds via catechol-O-methyltransferase, which is a known vasoactive compound [30]. 

Due to their synergistic action, certain combinations of polyphenol metabolites exert greater antihypertensive effects compared with individually. Quercetin derivates, such as 3,4-dihydroxyphenylacetic acid (DHPA), 4-methylcatechol (4MC), and 3-(3-hydroxyphenyl) propionic acid (3HPPA), show different hypotensive effects if they act together [31]. Despite the low availability observed for some polyphenols, vasodilator action is shown to occur through their metabolites at the colonical level. Through vasodilator effects on aortic vascular smooth muscle, 3HPPA is the compound with the highest efficacy, in whose mechanism of action research observed how the endothelium and nitric oxide (NO) played a role in vascular relaxation. [32]. 

Therefore, the synergistic effect may be attributed to the different mechanisms of action of each metabolite [33]. The mechanism of action of the polyphenols presented in the nutraceutical used in our study (catechins and epicatechins) could be an increase in endothelial nitric oxide synthase (ONSe), resulting in enhanced endothelium-dependent vasodilation [34]. Another mechanism of action that may explain the decrease in blood pressure is the possible inhibition of the angiotensin-converting enzyme and, thus, the reduction of angiotensin II and its hypertensive mechanism [35].

In addition, research showed that *H. sabdariffa* has a compound that causes nitric oxide release from the vascular endothelium followed by kidney filtration increase, a mechanism that clears its diuretic effect on the blood pressure [36,37]. Another study reported a significant decrease in the blood pressure of patients treated with HS, reporting differences between both the control group and the experimental group. However, a limitation of the study was that all subjects were encouraged to lose weight, as well as to control their sodium/potassium intake ratio, which could be partially responsible for the results observed [37]. 

In addition, researchers reported that the polyphenols derived from HS-LC extract might be able to increase the adiponectin gene expression and PPAR-α, while reducing the NF-kB protein, which regulates the genes that mediate immune and inflammatory responses [38,39]. Adiponectin has an anti-inflammatory capacity, leading to the activation of AMPK in hypertrophic adipocytes [39]. Together, these mechanisms may explain weight control and related pathologies as hypertension.

Finally, the morning has also been associated with a greater risk for cardiovascular events compared with the rest of the day, due to fluctuations in the blood pressure flow and hemostatic changes [40,41].

## 4. Materials and Methods

The study consisted of a double-blind, randomized, placebo-controlled clinical trial, with two parallel branches to study depending on the extract consumed (experimental or placebo) and single-center. A total of 80 pre-hypertensive or type 1 hypertensive subjects of both sexes were included in the study after matching all the including criteria (age between 18–65 years, systolic blood pressure higher than 120 mmHg, diastolic blood pressure higher than 80 mmHg, and no pharmacological treatment for hypertension) and none of the exclusion criteria (illness, other pharmacological treatment, toxicological habits, or allergies). 

After full disclosure of the implications and restrictions of the protocol, the subjects were required to sign an informed consent. The determination of blood pressure was done following the ESC/ESH criteria [5,42]. The study was conducted in accordance with the Declaration of Helsinki (randomized trial registration number (Clinicaltrials): NCT04105192).

The product under study (MetabolAid^®^) consisted of a capsule, including a mixture of 500 mg of HS-LC extracts (175 mg of HS and 325 mg of LC), leading to high quantities of verbascoside and anthocyanins, respectively [43]. The placebo capsules contained 500 mg of crystalline microcellulose, maintaining the same aspects as the product under study. MetabolAid^®^ was provided by Monteloeder S.L. (Alicante, Spain) (Patent application number WO2019058011A1). The polyphenolic composition of the product was quantified and reported in previous studies [17,43] that showed two main polyphenolic families (phenylpropanoids and anthocyanins). 

The total polyphenolic composition was represented by phenylpropanoids (16% of the total dry weight) and anthocyanins (3.5% of the total dry weight). Four main phenolic compounds were identified; two phenylpropanoids (verbascoside and isoverbascoside) and two anthocyanins (delphinidin-3-O-sambubioside and cyanidin-3-O-sambubioside). Regarding the phenylpropanoids, 15% corresponded to verbascoside (93.75% of the total of phenylpropanoids) and 1% to isoverascoside (representing 6.25%). For the anthocyanins, 2.27% corresponded to delphinidin-3-O-sambubioside and 1.23% to cyanidin-3-O-sambubioside representing 65% and 35% of the total anthocyanin content, respectively. 

The length of the present study was 84 days, during which the subjects consumed the botanical mixture or the placebo daily depending on the previous randomization. Therefore, 40 subjects were distributed in the placebo group, and the other 40 subjects were allocated in the HS-LC group. Each subject that joined the study was assigned a code (generated by a number software generator (Epidat v4.1)) assigning them to one of the two study groups. Both the researchers and the participants themselves did not know the group they belonged to.

**Figure 5:**
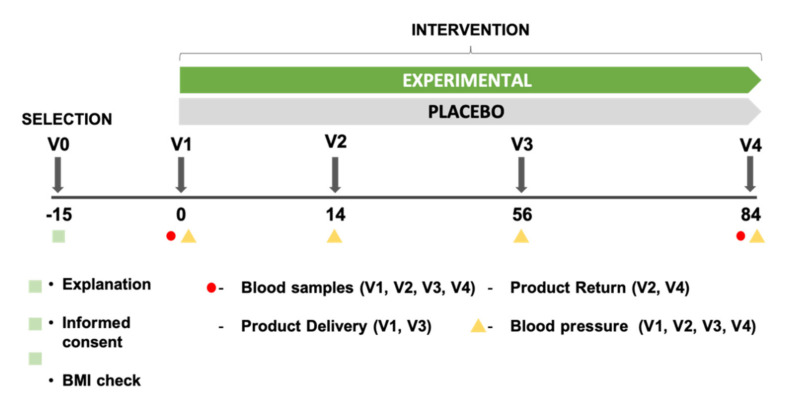
Graphical representation of the study.

As displayed in Figure 5, the subjects attended the research center at the beginning of the study and at the end. At baseline, blood sampling was obtained from the cubital vein of subjects from both groups. After blood collection, and after explaining the operation of the study, the subjects received a treatment based on the prior randomization. To determine the physical activity, every subject was equipped with an accelerometer (ActiGraph wGT3X-BT) prior to the beginning of the study. After 84 days of intake of the HS-LC extract or the placebo, the same protocol was repeated.

The blood pressure was determined using an oscillometer sphygmomanometer to determine the systolic and diastolic blood pressures, necessary for the inclusion criteria of the study [44]. However, the main limitations of this procedure are that it only offers information of the value at a specific time and that it has numerous biases [40]. In 2011, the British guides of the National Institute for Health and Clinical Excellence 9 and, subsequently, different scientific societies and institutions recommended performing an ABMP to confirm the diagnosis of HT [45] Therefore, the oscillometric sphygmomanometer was only used as a method for selecting study volunteers, and not as an analytical measure of the study.

The influence of the HS-LC extract on blood pressure was determined with an ABPM. The measurement of blood pressure was performed at baseline (prior to product consumption), at days 14, 56, and 84 from the beginning of the study. The ABPM method was done using a Holter Spacelabs Healthcare (holter) device [46]. The cuff was placed on the non-dominant arm so that the subject could comfortably follow their daily life activities. The holter was engaged at the waist by a belt. 

The volunteers were instructed to be quiet every time the cuff expanded. The holter was programmed to measure every hour for 24 h, and thus, after this time, the subjects returned the device. The values were displayed as the mean values of the measurements performed over 24 h, obtaining systolic and DBP determinations. Depending on the moment of the day, the daytime blood pressure and nighttime blood pressure were determined considering the morning hours and nighttime hours.

Other parameters related to blood pressure were also reported as the MBP that measures systemic irrigation providing information on the effectiveness with which blood reaches from the heart to the organs. The MBP was obtained from the values obtained along 24 h and as an hourly measurement, resulting from the following formula: MBP = DP + ((SBP-DBP)/3) [47,48].

Additionally, due to the importance of exercise on cardiovascular system, physical activity can be partly-determinant of the possible observed effects during the study. Therefore, in order to prove if some strategies are effective for the treatment of obesity, physical exercise must be measured and regulated. For this purpose, the metabolic equivalent of the task (MET) was determined throughout the whole length of the study. That fact led to the determination of metabolic equivalents that relates the intensity of physical activity with the kilocalories consumed by subjects, in order to standardize the exercise throughout the study. MET values were determined in accordance with the “Compendium of Physical Activities” [49].

### Statistic Determinations

Categorical variables were expressed as frequencies and percentages, and the continuous variables were expressed as the means and standard errors (SEs). The chi-square (χ2) test or the Fisher’s exact probability test was used for the comparison of categorical variables between the experimental and placebo groups. Quantitative variables were assessed using the analysis of variance (ANOVA) for repeated measures with two factors: time (baseline and final), as within-subject factors and intervention (experimental and placebo) as the between-subject factor with Bonferroni’s correction for pairwise comparisons. We considered 0.05 as the level of significance. Statistical analysis was carried out with the SPSS 21.0 software.

## 5. Conclusions

The daily consumption of the HS-LC extract but not the placebo over 84 days was able to reduce the daytime parameters related to blood pressure. These parameters were established as leading indicators of hypertensive patients, and their reduction is key for the treatment of hypertension and related pathologies. Specifically, the supplementation with HS-LC extract reduced the SBP and MBP as measured using ABMP. The most remarkable results were observed in the daily measurements and particularly in the SBP, which showed a descent of 3%. The nocturnal measurements were less noticeable but revealed that the supplementation with the HS-LC extract was able to reduce all the parameters under study.

## Figures and Tables

**Table 1:** Characteristics of the volunteers from the control and experimental (*Hibiscus sabdariffa* (HS) and *Lippia citriodora* (LC)) groups. Body mass index (BMI), metabolic equivalent of task (MET), systolic blood pressure (SBP), and diastolic blood pressure (DBP).

	Control	Hs-Lc Extract	*p*
Gender (number: % men)	33: 84.62%	25: 67.57%	0.107
Age (years)	31.51 ± 11.80	38.30 ± 12.02	0.015
BMI (kg/m^2^)	27.95 ± 5.98	28.86 ± 3.81	0.431
Physical activity (METs · min/week)	711.19 ± 111.81	745.69 ± 189.07	0.333
SBP Sphygmomanometer (mmHg)	133.64 ± 12.42	135.43 ± 11.44	0.516
DBP Sphygmomanometer (mmHg)	86.13 ± 7.60	90.27 ± 8.05	0.150

**Table 2:** The evolution of biochemical blood parameters of subjects during the study.

		Baseline	Final
Total Cholesterol (mg/dL)	Control	228.2 ± 6.2	232.8 ± 9.5
	Extract	234.9 ± 6	223.2 ± 9
HDL Cholesterol(mg/dL)	Control	56.5 ± 2.5	58.9 ± 2.4
	Extract	58.5 ± 2.1	60,6 ± 2.2
LDL Cholesterol (mg/dL)	Control	144.1 ± 6.3	144.1 ± 7.6
	Extract	134.1 ± 7.2	133.8± 7.5
Triglycerides (mg/dL)	Control	138.8 ± 12.1	134.1 ± 612.4
	Extract	136 ± 612.4	133.1 ± 12.4
Blood Glucose (mg/dL)	Control	97 ± 3.5	94.4 ± 2.8
	Extract	94.8 ± 2.9	93.2 ± 2.3
Glycated Hemoglobin (%)	Control	5.4 ± 0.2	5.2 ± 0.1
	Extract	5 ± 0.2	4.8 ± 0.1

## Data Availability

Not applicable.

## Sample Availability

Samples of the compounds are available from the authors.
